# Using AI to improve peer review and research integrity in scientific journals

**DOI:** 10.1093/haschl/qxag028

**Published:** 2026-02-07

**Authors:** Howard Bauchner, Frederick Rivara

**Affiliations:** Boston University Chobanian & Avedisian School of Medicine, Visiting Scholar, National University of Singapore; Department of Pediatrics, University of Washington School of Medicine, Seattle, WA 98195, United States

**Keywords:** peer review, research integrity, AI

## Abstract

Peer review is inefficient, biased, and often ineffective. However, its importance in maintaining research integrity, at a time when the public has less faith in science, is clear, since journals are the principal conduit for communicating the results of research to the scientific community and the public. Given that the number of published manuscripts in the biomedical sciences now exceeds 3 million per year it is no longer possible for human editorial and peer review alone to ensure integrity. New approaches are needed, including the use of artificial intelligence (AI) to assist in editorial and peer review.

It is well known that human editorial and peer review is inefficient, biased, and often ineffective. In addition, there is growing concern about the public's increasing lack of trust in science, reflecting, in part, the challenges of maintaining and improving research integrity. As defined by the UK Research Integrity Office, research integrity refers to “all of the factors that underpin good research practice and promote trust and confidence in the research process,” including rigor, honesty, respect, and transparency.^1^

Journals play a key role in maintaining research integrity since they are the principal conduit for communicating the results of research to the scientific community and the public. Given that the annual number of published manuscripts in the biomedical literature now exceeds 3 million, with many more papers submitted but rejected, it is no longer possible for human editorial and peer review alone to assure research integrity. The time has come to commit to artificial intelligence (AI) to assist in this process. Interestingly, a recent survey of 1645 scientists indicated that 53% (of 909) acknowledged using large language models (LLMs) when asked to review manuscripts, despite instructions from some journals that these models should not be used.^2^

Maintaining research integrity has many challenges, including evidence of data-dredging, observational data that can be analyzed to “find” virtually any result, the subjective interpretation of evidence, and massive amounts of data now available for analysis.^3-5^ These challenges, some of which AI can help to ameliorate, require a renewed commitment by the research community to maintain research integrity.

In this commentary, we suggest the following 5 actions that are needed to improve journals’ ability to ensure research integrity by using AI to assist in manuscript review.

1. The reporting of observational data needs to change. It is clear, given the many assumptions that are made in analyzing data, that different outcomes can be produced from the same dataset. For example, using specification curve analysis, derived from a systematic review of articles reporting the relationship between red meat and mortality, Wang et al^4^ identified 1208 analytic approaches. When applied to the National Health and Nutrition Examination Survey (NHANES) dataset, 435 analyses produced a hazard ratio greater than 1 (implying increased mortality) and 773 a hazard ratio less than 1. It is time for journals to require the registration of observational cohort studies, including a statistical analytic plan and protocol, particularly those that are attempting to assess causal relations or emulate clinical trials with real-world data. Registration of randomized controlled trials (RCTs) prior to data collection along with an accompanying statistical analytic plan has transformed reporting of RCTs. Data dredging and manipulation of the primary outcome in RCTs are no longer possible. It should be acknowledged that it will be very difficult for any single journal to commit to registration of observational cohort studies without others doing the same; otherwise, that journal would be at a competitive disadvantage. The success of registration of RCTs was, in part, because this was endorsed by the International Committee of Medical Journal Editors (ICMJE), with all major clinical journals requiring registration beginning on a specific date. If a similar change could be implemented for observational cohort studies, the ability of journals to assess research integrity would be enhanced. If there is a commitment to register observational cohort studies, AI could be used to assess the accuracy of registration.
2. There is broad agreement that RCTs and meta-analyses should not be published unless they are registered prior to data collection; yet, for both study types, journals continue to publish unregistered studies. It is time for greater enforcement of this requirement, which AI could assess. If journals do publish such studies that have not been appropriately registered, they should indicate why this was done. Some have challenged the commitment to publish all RCTs, given that some are not appropriately registered or conducted, but investigators have an ethical responsibility to make the results of RCTs publicly available.
3. The number needed to treat (NNT) and number needed to harm should be reported for RCTs and observational studies when feasible. When the media report that a trial was “successful,” we suspect that many people believe that most participants benefited from treatment—that is not true and is misleading. For example, a recent study examined outcomes in a group of 295 971 veterans, 164 132 who recently received COVID-19 and influenza vaccines compared with 131 839 who only received influenza vaccine.^6^ The COVID-19 vaccine was 64% effective in reducing COVID-19 reported mortality, with a NNT of 4545. Clearly, these 2 “outcomes,” while accurate, are likely to be interpreted differently by some people. Although the NNT can be a difficult concept to explain, it can enhance the conversation between a clinician and patient when they are discussing potential treatment options. AI could quite easily calculate the NNT when appropriate.
4. Many journals require that authors and peer-reviewers highlight the appropriate reporting guideline and whether it was followed. Other than CONSORT (Consolidated Standards of Reporting Trials) and PRISMA (Preferred Reporting Items for Systematic Reviews and Meta-Analyses), we are unaware of any data suggesting that peer-reviewers check if this was done, despite the request by journal editors. AI has already been used to assess adherence to CONSORT and could easily be used to assess adherence to appropriate guidelines.
5. Other challenges have emerged—for example, the accuracy of references in manuscripts, letters to the editor written by AI, the persistent concern of image manipulation, and manuscripts written by AI but not declared by authors, despite recommendations from ICMJE and other groups. Each of these issues can be evaluated by AI.

Funders, federal agencies, and others also have a role to play in ensuring research integrity—including greater transparency. For example, the National Institutes of Health (NIH) has been lax in enforcing its mandate that the results of NIH-supported RCTs be posted on clinicaltrials.gov. Time will tell if it is more effective at enforcing the new mandate of data sharing. It recently announced a renewed commitment to reproducibility and replicability, but an appropriate reward system has not yet emerged. The Food and Drug Administration (FDA) has a treasure trove of data about the approval of all drugs and devices. Indeed, they have individual patient data, yet these are unavailable for re-analysis. This, despite concerns raised by Secretary Robert Kennedy about the safety of mRNA COVID-19 vaccines.

To assist with the changes suggested previously, AI should now be used alongside human editorial and peer review. Data have emerged that AI is effective, can identify adherence to CONSORT requirements, and assess if appropriate statistical analyses were performed.^7-10^

It is likely in the future that AI will be able to assess adherence with most of the reporting guidelines, determine if RCTs and meta-analyses have been registered and are being reported consistent with registration, check references for accuracy, help identify appropriate peer-reviewers, assess if potential peer-reviewers as suggested by the authors are appropriate, determine if AI was used in writing the manuscript or letters to the editor (and if that was declared by the authors, as now requested by many journals), and check for image manipulation—all tasks that are important to ensure research integrity and that are nearly impossible for most journals to do, even those that are well-funded.

Human review should not be abandoned, but rather AI should be used as an adjunct to peer review, just as plagiarism software is now used at many journals. Prior to review by an editor, assuming a manuscript is considered in scope and meets basic requirements, AI review could be performed. Following that review, an editor could than decide if the manuscript is worthy of external peer review, or it could be returned to the author with initial comments for revision or rejected. Recently, *NEJM AI* editorialized about their use of AI in conjunction with human peer review—commenting on its successes and challenges.^11^

There are concerns about harnessing AI to assist in manuscript review. Most important is the protection of the data submitted by authors. Whatever LLM is used, the data underpinning the manuscript must be considered confidential, and not used by the LLM for any purpose. In addition, just as human peer review can be biased, so too could AI review—hence, the importance of a collaborative relationship between humans and LLMs.

It is time for journals to consider modifying their instructions to peer reviewers if they have indicated that LLMs should not be used since clearly they are using such tools. In the survey referenced previously, the percentage of peer-reviewers acknowledging use of LLMs increased by 24% in 1 year.^2^ Certainly, if the journal is using LLMs to assist in peer review they should allow peer-reviewers to use it. Rather than having “ghost” peer-reviewers, similarly to “ghost” authors, by modifying their instructions to peer-reviewers, the use of LLMs will be transparent.

Public trust in science, especially in the United States, is waning. Given the role that journals play in the ecosystem of science, it is important that editorial integrity be maintained and even enhanced. It is no longer possible for human editorial and peer review alone to effectively assure research integrity. New policies and approaches are needed, including the use of AI to assist in manuscript review.

## Supplementary Material

qxag028_Supplementary_Data

## References

[1] K Research Integrity Office. Home page. Accessed January 11, 2026. https://ukrio.org/

[2] Accessed December 18, 2025. https://www.frontiersin.org/documents/unlocking-ai-potential.pdf

[3] Spick M, Onoja A, Harrison C, et al Quantifying new threats to health and biomedical literature integrity from rapidly scaled publications and problematic research. medRxiv 25331008. 10.1101/2025.07.07.25331008, July 9, 2025, preprint: not peer reviewed. PMC13178217 41740900

[4] Wang Y, Pitre T, Wallach JD, et al Grilling the data: application of specification curve analysis to red meat and all-cause mortality. J Clin Epidemiol. 2024;168:111278. 10.1016/j.jclinepi.2024.111278 38354868 PMC12289182

[5] Bauchner H, Ioannidis JPA. The subjective interpretation of the medical evidence. JAMA Health Forum. 2024;5(3):e240213. 10.1001/jamahealthforum.2024.0213 38551587

[6] Cai M, Xie Y, Al-Aly Z. Association of 2024-2025 COVID-19 vaccine with COVID-19 outcomes in U.S. veterans. N Engl J Med. 2025;393(16):1612–1623. 10.1056/NEJMoa2510226 41061231

[7] Liang W, Zhang Y, Cao H, et al Can large language models provide useful feedback on research papers? A large-scale empirical analysis. NEJM AI. 2024;1(8). 10.1056/AIoa2400196

[8] Alahab F, Franco J, Macdonald H, Schroter S. Quality and comprehensiveness of peer reviews of journal submissions produced by large language models vs humans. Accessed January 3, 2026. https://peerreviewcongress.org/abstract/quality-and-comprehensiveness-of-peer-reviews-of-journal-submissions-produced-by-large-language-models-vs-humans/

[9] Wrightson JG, Blazey P, Moher D, et al GPT for RCTs? Using AI to determine adherence to clinical trial reporting guidelines. BMJ Open. 2025;15(3):e088735. 10.1136/bmjopen-2024-088735. PMC11927406 40107689

[10] Bruns STB, Herwartz H, Ioannidis JA, et al Automated interpretation of statistical tables to assess reporting errors and associations with open science policies in economic journals. Accessed January 3, 2026. https://peerreviewcongress.org/abstract/automated-interpretation-of-statistical-tables-to-assess-reporting-errors-and-associations-with-open-science-policies-in-economics-journals/

[11] Manrai KR, Ouyang D, Hogan JW, Kohan IS. Accelerating science with human + AI review. NEJM AI. 2025;2(12). 10.1056/AIe2501175.

